# Supplementary material for: Nondiffracting supertoroidal pulses and optical “Kármán vortex streets”
Source: Nat Commun. 2024 Jun 7;15:4863. doi: 10.1038/s41467-024-48927-5 (PMC11161654; doi:10.1038/s41467-024-48927-5)
Supplement: Supplementary file 1 — Supplementary Information [file 41467_2024_48927_MOESM1_ESM.pdf]

Supplementary Information for:

Nondiffracting supertoroidal pulses and optical Kármán vortex streets

Yijie Shen,[1, 2, *] Nikitas Papasimakis,[3] and Nikolay I. Zheludev[1, 3]

[1] Centre for Disruptive Photonic Technologies, School of Physical and Mathematical Sciences and The Photonics Institute, Nanyang Technological University, Singapore 637378, Singapore
[2] School of Electrical and Electronic Engineering, Nanyang Technological University, Singapore 639798, Singapore
[3] Optoelectronics Research Centre & Centre for Photonic Metamaterials, University of Southampton, Southampton SO17 1BJ, United Kingdom

** Electronic address: yijie.shen@ntu.edu.sg*

Note 1: Derivation of nondiffracting supertoroidal pulses.

We start from the scalar generating function derived from modified power spectrum method [1,2]:

$$f = f_0 \frac{e^{-s/q_3}}{(q_1 + i\tau)(s + q_2)^{\alpha}} \tag{S1}$$

Where $s \equiv r^2/(q_1 + i\tau) - i\sigma$, $\tau = z - ct$, $\sigma = z + ct$, $r = \sqrt{x^2 + y^2}$, $c = 1/\sqrt{\mu_0\varepsilon_0}$ is the speed of light, the parameters q_1, q_2, q_3 are real positive with units of length, and the real dimensionless parameter α must satisfy $\alpha \ge 1$ in order for the electromagnetic pulse to fulfill finite energy. In conventional method, it always assumes that $q_3 \to \infty$ and $\alpha = 1$. Here we break the limits that the parameter α can be any real number no less than one and q_3 can be any value in length dimension. The azimuthally polarized pulse is derived under a curled vector Hertz potential $\mathbf{\Pi} = \nabla \times \hat{\mathbf{z}} f(\mathbf{r}, t)$ in cylindrical coordinate (r, θ, z), the TE-mode electromagnetic field can be generated from Hertz potential by:

$$\begin{cases} \mathbf{E}(\mathbf{r},t) = -\mu_0 \dfrac{\partial}{\partial t} \nabla \times \mathbf{\Pi} = \hat{\boldsymbol{\theta}} \mu_0 \partial_r \partial_t f \\ \mathbf{H}(\mathbf{r},t) = \nabla \times (\nabla \times \mathbf{\Pi}) = \hat{\mathbf{r}} \partial_r \partial_z f + \hat{\mathbf{z}} \left(\partial_z^2 - \dfrac{1}{c^2} \partial_t^2 \right) f \end{cases} \tag{S2}$$

Electric field: Based on equation set (S2), the amplitude of azimuthally polarized electric field can be derived as:

$$\begin{aligned} E_\theta &= \mu_0 \partial_z \partial_t f = \mu_0 f_0 \partial_\rho \partial_t \frac{e^{-s/q_3}}{(q_1 + i\tau)(s + q_2)^{\alpha}} \\ &= \mu_0 f_0 2ice^{-s/q_3} \left[\frac{(\alpha+1)\alpha ru}{(q_1+i\tau)^2 (q_2+s)^{\alpha+2}} - \frac{2\alpha r}{(q_1+i\tau)^3 (q_2+s)^{\alpha+1}} + \frac{2\alpha ru}{q_3 (q_1+i\tau)^2 (q_2+s)^{\alpha+1}} \right. \\ &\quad \left. - \frac{2r}{q_3 (q_1+i\tau)^3 (q_2+s)^{\alpha}} - \frac{ru}{q_3^2 (q_1+i\tau)^2 (q_2+s)^{\alpha}} \right] \\ &= \frac{2i\mu_0 f_0 cre^{-s/q_3}}{(q_1+i\tau)^2 (q_2+s)^{\alpha}} \left[\frac{(\alpha+1)\alpha u(q_1+i\tau) - 2\alpha(q_2+s)}{(q_1+i\tau)(q_2+s)^2} + \frac{2\alpha u(q_1+i\tau) - 2(q_2+s)}{q_3(q_1+i\tau)(q_2+s)} - \frac{u}{q_3^2} \right] \end{aligned} \tag{S3}$$

where $u = r^2/(q_1 + i\tau)^2 - 1$. When $q_3 \to \infty$ and $\alpha = 1$, the equation (S3) will be reduced into:

$$E_\theta \big|_{\alpha=1, q_3 \to \infty} = 4i\mu_0 f_0 cr \frac{u(q_1+i\tau) - (q_2+s)}{(q_1+i\tau)^3 (q_2+s)^3} \tag{S4}$$

Then we prove that the equation (S4) is exactly the expression of the azimuthal electric field of fundamental flying doughnut. Substitute $u = r^2/(q_1 + i\tau)^2 - 1$, $s \equiv r^2/(q_1 + i\tau) - i\sigma$, $\tau = z - ct$, $\sigma = z + ct$ into equation (S4), we get:

$$
\begin{aligned}
E_\theta &= 4i\mu_0 f_0 cr \frac{\left[\dfrac{r^2}{(q_1+i\tau)^2}-1\right](q_1+i\tau)-\left(q_2+\dfrac{r^2}{q_1+i\tau}-i\sigma\right)}{(q_1+i\tau)^3\left(q_2+\dfrac{r^2}{q_1+i\tau}-i\sigma\right)^3} \\
&= 4i\mu_0 f_0 cr \frac{\left[r^2-(q_1+i\tau)^2\right]-\left[r^2+(q_1+i\tau)(q_2-i\sigma)\right]}{(q_1+i\tau)\left[r^2+(q_1+i\tau)(q_2-i\sigma)\right]^3} \\
&= 4i\mu_0 f_0 cr \frac{-(q_1+i\tau)-(q_2-i\sigma)}{\left[r^2+(q_1+i\tau)(q_2-i\sigma)\right]^3} = -4if_0\mu_0 c\frac{r(q_1+q_2-2ict)}{\left[r^2+(q_1+i\tau)(q_2-i\sigma)\right]^3}
\end{aligned}
\tag{S5}
$$

This equation (S5) is exactly the expression of the electric field of the fundamental flying doughnut pulse [2].

Magnetic field: The magnetic field of the general transversely divergent pulse includes the radial and longitudinal components, based on equation set (S2), the radial component can be derived as

$$
\begin{aligned}
H_r &= \partial_r\partial_z f = f_0\partial_r\partial_z \frac{e^{-s/q_3}}{(q_1+i\tau)(s+q_2)^\alpha} \\
&= \frac{2if_0 re^{-s/q_3}}{(q_1+i\tau)^2(q_2+s)^\alpha}\left[\frac{-(\alpha+1)\alpha v}{(q_2+s)^2}+\frac{2\alpha}{(q_1+i\tau)(q_2+s)}-\frac{2\alpha v}{q_3(q_2+s)}+\frac{2}{q_3(q_1+i\tau)}-\frac{v}{q_3^2}\right] \\
&= \frac{2if_0 re^{-s/q_3}}{(q_1+i\tau)^2(q_2+s)^\alpha}\left[\frac{2\alpha(q_2+s)-(\alpha+1)\alpha v(q_1+i\tau)}{(q_1+i\tau)(q_2+s)^2}+\frac{2(q_2+s)-2\alpha v(q_1+i\tau)}{q_3(q_1+i\tau)(q_2+s)}-\frac{v}{q_3^2}\right]
\end{aligned}
\tag{S6}
$$

where $v = r^2/(q_1+i\tau)^2+1$. When $q_3 \to \infty$ and $\alpha = 1$, the equation (S6) will be reduced into:

$$
H_r\big|_{\alpha=1,q_3\to\infty} = 4if_0 r\frac{(q_2+s)-v(q_1+i\tau)}{(q_1+i\tau)^3(q_2+s)^3} \tag{S7}
$$

Then we prove that the equation (S7) is exactly the radial magnetic field of fundamental flying doughnut. Substitute $v = r^2/(q_1+i\tau)^2+1$, $s \equiv r^2/(q_1+i\tau)-i\sigma$, $\tau = z-ct$, $\sigma = z+ct$ into equation (S7), we get:

$$
\begin{aligned}
H_r &= 4if_0 r \frac{\left(q_2+\dfrac{r^2}{q_1+i\tau}-i\sigma\right)-\left[\dfrac{r^2}{(q_1+i\tau)^2}+1\right](q_1+i\tau)}{(q_1+i\tau)^3\left(q_2+\dfrac{r^2}{q_1+i\tau}-i\sigma\right)^3} \\
&= 4if_0 r \frac{\left[r^2+(q_1+i\tau)(q_2-i\sigma)\right]-\left[r^2+(q_1+i\tau)^2\right]}{(q_1+i\tau)\left[r^2+(q_1+i\tau)(q_2-i\sigma)\right]^3} \\
&= 4if_0 r \frac{(q_2-i\sigma)-(q_1+i\tau)}{\left[r^2+(q_1+i\tau)(q_2-i\sigma)\right]^3} = 4if_0 r\frac{r(q_2-q_1-2iz)}{\left[r^2+(q_1+i\tau)(q_2-i\sigma)\right]^3}
\end{aligned}
\tag{S8}
$$

This equation (S8) is exactly the closed-form expression of the radial magnetic field of the fundamental toroidal pulse [2].

The longitudinal component of magnetic field can be divided into two terms as follow:

$$\partial_z^2 f = f_0 \partial_z^2 \frac{e^{-s/q_3}}{(q_1+i\tau)(s+q_2)^\alpha}$$

$$= \frac{f_0 e^{-s/q_3}}{(q_1+i\tau)(q_2+s)^{\alpha+2}} \left[-(1+\alpha)\alpha v^2 + \frac{2\alpha v(q_2+s)}{q_1+i\tau} + \frac{2\alpha r^2(q_2+s)}{(q_1+i\tau)^3} - \frac{2(q_2+s)^2}{(q_1+i\tau)^2} - \frac{2\alpha v^2(q_2+s)}{q_3} + \frac{2v(q_2+s)^2}{q_3(q_1+i\tau)} + \frac{2r^2(q_2+s)^2}{q_3(q_1+i\tau)^3} - \frac{v^2(q_2+s)^2}{q_3^2} \right] \tag{S9}$$

$$-\frac{1}{c^2}\partial_t^2 f = -\frac{f_0}{c^2}\partial_t^2 \frac{e^{-s/q_3}}{(q_1+i\tau)(s+q_2)^\alpha}$$

$$== -\frac{f_0 e^{-s/q_3}}{(q_1+i\tau)(q_2+s)^{\alpha+2}} \left[-(1+\alpha)\alpha u^2 + \frac{2\alpha u(q_2+s)}{q_1+i\tau} + \frac{2\alpha r^2(q_2+s)}{(q_1+i\tau)^3} - \frac{2(q_2+s)^2}{(q_1+i\tau)^2} - \frac{2\alpha u^2(q_2+s)}{q_3} + \frac{2u(q_2+s)^2}{q_3(q_1+i\tau)} + \frac{2r^2(q_2+s)^2}{q_3(q_1+i\tau)^3} - \frac{u^2(q_2+s)^2}{q_3^2} \right] \tag{S10}$$

Then the longitudinal component of magnetic field can be derived as the sum of equations (S9) & (S10):

$$H_z = \left(\partial_z^2 - \frac{1}{c^2}\partial_t^2\right) f$$

$$= \frac{f_0 e^{-s/q_3} \left[(1+\alpha)\alpha\left(u^2-v^2\right) + \frac{2\alpha(v-u)(q_2+s)}{q_1+i\tau} - \frac{2\alpha\left(v^2-u^2\right)(q_2+s)}{q_3} + \frac{2(v-u)(q_2+s)^2}{q_3(q_1+i\tau)} - \frac{\left(v^2-u^2\right)(q_2+s)^2}{q_3^2} \right]}{(q_1+i\tau)(q_2+s)^{\alpha+2}} \tag{S11}$$

Where $u = r^2/(q_1+i\tau)^2 - 1$ and $v = r^2/(q_1+i\tau)^2 + 1$, thus $v-u=2$ and $v^2-u^2=(v-u)(v+u)=4r^2/(q_1+i\tau)^2$, then the equation (S11) can be further simplified as:

$$H_z = \frac{f_0 e^{-s/q_3} \left[-\frac{(1+\alpha)\alpha 4r^2}{(q_1+i\tau)^2} + \frac{4\alpha(q_2+s)}{q_1+i\tau} - \frac{8\alpha r^2(q_2+s)}{q_3(q_1+i\tau)^2} + \frac{4(q_2+s)^2}{q_3(q_1+i\tau)} - \frac{4r^2(q_2+s)^2}{q_3^2(q_1+i\tau)^2} \right]}{(q_1+i\tau)(q_2+s)^{\alpha+2}}$$

$$= -\frac{4f_0 e^{-s/q_3}}{(q_1+i\tau)^3(q_2+s)^{\alpha+2}} \left[(1+\alpha)\alpha r^2 - \alpha(q_1+i\tau)(q_2+s) + \frac{2\alpha r^2(q_2+s) - (q_1+i\tau)(q_2+s)^2}{q_3} + \frac{r^2(q_2+s)^2}{q_3^2} \right] \tag{S12}$$

When $q_3 \to \infty$ and $\alpha = 1$, the equation (S12) will be reduced into:

$$H_z\big|_{\alpha=1, q_3\to\infty} = -4f_0 \frac{2r^2 - (q_1+i\tau)(q_2+s)}{(q_1+i\tau)^3(q_2+s)^3} \tag{S13}$$

Substitute $s \equiv r^2/(q_1+i\tau) - i\sigma$, $\tau = z - ct$, $\sigma = z + ct$ into equation (S13), we get:

$$H_z\big|_{\alpha=1,q_3\to\infty} = -4f_0\frac{2r^2-(q_1+i\tau)\left(q_2+\frac{r^2}{q_1+i\tau}-i\sigma\right)}{(q_1+i\tau)^3\left(q_2+\frac{r^2}{q_1+i\tau}-i\sigma\right)^3}$$
$$= -4f_0\frac{2r^2-\left[r^2+(q_1+i\tau)(q_2-i\sigma)\right]}{\left[r^2+(q_1+i\tau)(q_2-i\sigma)\right]^3} = -4f_0\frac{r^2-(q_1+i\tau)(q_2-i\sigma)}{\left[r^2+(q_1+i\tau)(q_2-i\sigma)\right]^3} \quad \text{(S14)}$$

This equation (S14) is exactly the closed-form expression of the longitudinal magnetic field of the fundamental flying doughnut pulse [2].

Electromagnetic field: In summary, combining equations (S3), (S6), and (S12), the electromagnetic field of the general transversely divergent pulse related to q_1, q_2, q_3, and α can be given by.

$$\begin{cases} E_\theta = \dfrac{2i\mu_0 f_0 c r e^{-s/q_3}}{(q_1+i\tau)^2(q_2+s)^\alpha}\left[\dfrac{(\alpha+1)\alpha u(q_1+i\tau)-2\alpha(q_2+s)}{(q_1+i\tau)(q_2+s)^2}+\dfrac{2\alpha u(q_1+i\tau)-2(q_2+s)}{q_3(q_1+i\tau)(q_2+s)}-\dfrac{u}{q_3^2}\right] \\ H_r = \dfrac{2if_0 r e^{-s/q_3}}{(q_1+i\tau)^2(q_2+s)^\alpha}\left[\dfrac{2\alpha(q_2+s)-(\alpha+1)\alpha v(q_1+i\tau)}{(q_1+i\tau)(q_2+s)^2}+\dfrac{2(q_2+s)-2\alpha v(q_1+i\tau)}{q_3(q_1+i\tau)(q_2+s)}-\dfrac{v}{q_3^2}\right] \\ H_z = -\dfrac{4f_0 e^{-s/q_3}}{(q_1+i\tau)^3(q_2+s)^{\alpha+2}}\left[(1+\alpha)\alpha r^2-\alpha(q_1+i\tau)(q_2+s)+\dfrac{2\alpha r^2(q_2+s)-(q_1+i\tau)(q_2+s)^2}{q_3}+\dfrac{r^2(q_2+s)^2}{q_3^2}\right] \end{cases} \quad \text{(S15)}$$

When $q_3 \to \infty$ and $\alpha = 1$, the electromagnetic field of general transversely divergent pulse will be reduced into that of the fundamental toroidal pulse [2]:

$$\begin{cases} E_\theta = -4if_0\mu_0 c\dfrac{r(q_1+q_2-2ict)}{\left[r^2+(q_1+i\tau)(q_2-i\sigma)\right]^3} \\ H_r = 4if_0\dfrac{r(q_2-q_1-2iz)}{\left[r^2+(q_1+i\tau)(q_2-i\sigma)\right]^3} \\ H_z = -4f_0\dfrac{r^2-(q_1+i\tau)(q_2-i\sigma)}{\left[r^2+(q_1+i\tau)(q_2-i\sigma)\right]^3} \end{cases} \quad \text{(S16)}$$

When $q_3 \to q_1$ and $\alpha = 1$, the electromagnetic field of general transversely divergent pulse will be reduced into that of the nondiffracting supertoroidal pulse, see details in Note 2.

***q_3* induced transverse divergence:** The electric field at focus for $q_3 = q_1 = 1$, $t = z = 0$ and $\alpha = 1$ is:

$$E_\theta = \frac{re^{-r^2/q_3}}{q_3^2\left(q_2+\frac{r^2}{q_3}\right)}\left[\frac{1-\frac{r^2}{q_3}}{q_3^2}+\frac{2q_3\left(\frac{r^2}{q_3}-1\right)-2\left(q_2+\frac{r^2}{q_3}\right)}{q_3\left(q_2+\frac{r^2}{q_3}\right)^2}+\frac{2q_3\left(\frac{r^2}{q_3}-1\right)-2\left(q_2+\frac{r^2}{q_3}\right)}{q_3^2\left(q_2+\frac{r^2}{q_3}\right)}\right] \quad \text{(S17)}$$

When $q_2 \gg q_3$ and assuming that we are only interested in $r \ll q_2$ due to the localization of energy, then $q_2 + r^2 / q_3 \approx q_2$, and the electric field can be simplified as:

$$E_\theta = \frac{re^{-r^2/q_3}}{q_3^2 q_2}\left[\frac{1-\dfrac{r^2}{q_3}}{q_3^2} + \frac{2q_3\left(\dfrac{r^2}{q_3}-1\right)-2q_2}{q_3 q_2^2} + \frac{2q_3\left(\dfrac{r^2}{q_3}-1\right)-2q_2}{q_3^2 q_2}\right]$$
$$= -\frac{re^{-r^2/q_3}}{q_3^6 q_2^3}\left[2q_3^2\left(q_3^2 - r^2\right) + q_2^2\left(q_3^2 + r^2\right) + q_2\left(4q_3^3 - 2q_3 r^2\right)\right] \tag{S18}$$

Since q_2 is very large, we can neglect the higher-order small terms with nominator of q_2^2, then the electric field can be further simplified as:

$$E_\theta(r)\Big|_{z=t=0} = \frac{re^{-r^2/q_3}(q_3^2 + r^2)}{q_3^6 q_2^3} \tag{S19}$$

Which has a peak value at $r = q_3$ (numerically $r = 0.96q_3$). Therefore, it shows that q_3 controls the transverse size of the pulse at focus.

Note 2: Theoretical proving of nondiffraction.

Hereinafter, we prove that the supertoroidal pulse as Eq. (S15) when $q_3 = q_1$ and $\alpha = 1$ fulfills the classic definition of nondiffraction:

$$\left|E(r,\theta,z,t)\right| = \left|E(r,\theta,z+\Delta z,t+\Delta z/c)\right| \tag{S20}$$

Where Δz is a given propagation distance. To prove this, we only need to prove the generating function, Eq. (S1), fulfilling the nondiffraction relationship:

$$\left|f(r,\theta,z,t)\right| = \left|f(r,\theta,z+\Delta z,t+\Delta z/c)\right| \tag{S21}$$

when $q_3 = q_1$ and $\alpha = 1$, substituting the relations of $s \equiv r^2/(q_1+i\tau) - i\sigma$, $\tau = z-ct$, $\sigma = z+ct$, and $q_2 \gg q_1$, the generating function can be written as:

$$\begin{aligned} f &= f_0 \frac{e^{-s/q_1}}{(q_1+i\tau)(s+q_2)} = f_0 \frac{e^{-\frac{r^2}{q_1(q_1+i\tau)}+\frac{i\sigma}{q_1}}}{(q_1+i\tau)\left(\frac{r^2}{q_1+i\tau}-i\sigma+q_2\right)} = f_0 \frac{e^{-\frac{r^2(q_1-i\tau)}{q_1(q_1^2+\tau^2)}+\frac{i\sigma}{q_1}}}{(q_2-i\sigma)\left[\frac{r^2}{q_2-i\sigma}+(q_1+i\tau)\right]} \\ &= f_0 \frac{e^{-\frac{r^2}{q_1^2+\tau^2}+i\frac{\tau r^2+\sigma(q_1^2+\tau^2)}{q_1(q_1^2+\tau^2)}}}{(q_2-i\sigma)\left[q_1+\frac{r^2(q_1+i\sigma)}{q_2^2+\sigma^2}+i\tau\right]} = f_0 \frac{e^{-\frac{r^2}{q_1^2+\tau^2}}e^{i\frac{\tau r^2+\sigma(q_1^2+\tau^2)}{q_1(q_1^2+\tau^2)}}}{(q_2-i\sigma)\left[q_1+\frac{q_1 r^2}{q_2^2+\sigma^2}+i\left(\frac{\sigma r^2}{q_2^2+\sigma^2}+\tau\right)\right]} \end{aligned} \tag{S22}$$

Because the assumptions of $q_2 \gg q_1$ and the radial distribution of the wave function is effectively confined in a finite region, the term $\frac{q_1 r^2}{q_2^2+\sigma^2} = \frac{q_1}{q_2}\frac{r^2}{q_2+\sigma^2/q_2} \doteq 0$ can be neglected. Because $\tau = z-ct$, $\sigma = z+ct$, and the pulse has an finite duration, which is much smaller than q_2/c (pulse width is much smaller than q_2). Therefore, the intensity of the generating function fulfills:

$$\left|f(r,\theta,z,t)\right|^2 = f_0^2 \frac{e^{-\frac{2r^2}{q_1^2+\tau^2}}}{\left(q_2^2+\sigma^2\right)\left[q_1^2+\left(\frac{\sigma r^2}{q_2^2+\sigma^2}+\tau\right)^2\right]} \tag{S23}$$

When $z = ct$, i.e. $\tau = 0$, the exponential term $e^{-2r^2/(q_1^2+\tau^2)}$ rapidly tend to zero for $r > q_1$, thus the radially dependent term in $\left(\frac{\sigma r^2}{q_2^2+\sigma^2}+\tau\right)^2$ can be neglected. In addition, the function $\frac{1}{q_2^2+\sigma^2}$ is a slowly variant function when $|\sigma| < q_2$, and the value of q_2 is much larger than the pulse duration. Therefore, this term works as a constant, and Eq. (S23) can be derived as:

$$\left|f(r,\theta,z,t)\right| \approx f_0 \frac{e^{-\frac{r^2}{q_1^2+\tau^2}}}{\sqrt{q_1^2+\tau^2}} = f_0 \frac{e^{-\frac{r^2}{q_1^2+(z-ct)^2}}}{\sqrt{q_1^2+(z-ct)^2}} \tag{S24}$$

Therefore, the nondiffraction relation, Eq. (S21), can be verified by:

$$\left|f(r,\theta,z+\Delta z,t+\Delta z/c)\right| = f_0 \frac{e^{-\frac{r^2}{q_1^2+[z+\Delta z-c(t+\Delta z/c)]^2}}}{\sqrt{q_1^2+\left[z+\Delta z-c(t+\Delta z/c)\right]^2}} = f_0 \frac{e^{-\frac{r^2}{q_1^2+(z-ct)^2}}}{\sqrt{q_1^2+(z-ct)^2}} = \left|f(r,\theta,z,t)\right| \qquad \text{(S25)}$$

The generating function is a nondiffracting signal and the supertoroidal pulse is nondiffracting. Supplementary Fig. 1 shows the simulated distributions of the real oscillation and amplitude of the supertoroidal pulse of $q_3 = q_1$, $q_2 = 100q_1$, and $\alpha = 1$, and the amplitude distribution given by Eq. (S24) at three different times upon propagation, where they show good agreement.

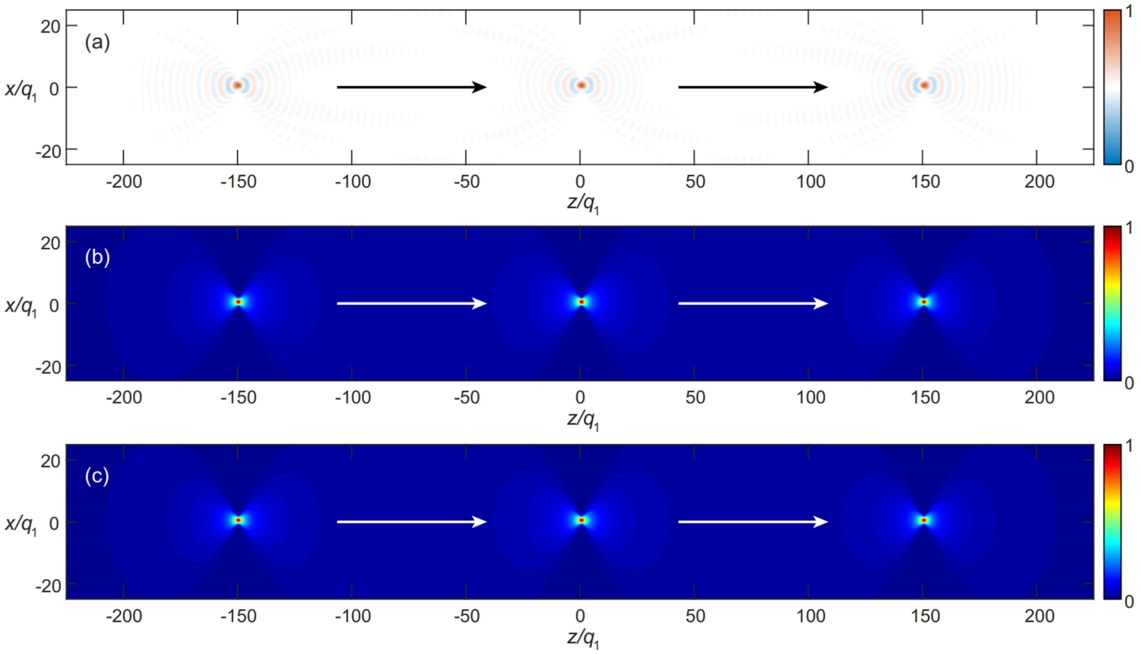

Supplementary Figure 1 (a) The simulated real oscillation signal and (b) amplitude distribution of the supertoroidal pulse of $q_3 = q_1$, $q_2 = 100q_1$, and $\alpha = 1$, given by Eq. (S15), and (c) the amplitude distribution given by Eq. (S21) at three different times $t = -150q_1/c$, $t = 0$, and $t = 150q_1/c$ upon propagation.

The diffraction properties of electromagnetic beams are typically quantified by the beam parameter. For Gaussian beams, the beam parameter is defined as the product of the divergence angle, θ, and the waist size, w_0. In this case, the beam parameter is a constant with value $\theta w_0=\lambda/\pi$. For the fundamental toroidal pulse, there is a similar relationship between the divergence and waist size [3], i.e. the beam size $w_0 = \sqrt{q_1 z_0}$, where q_1 is related to the central wavelength, $z_0=q_2/2$ is the Rayleigh range. For non-diffracting, finite energy, Bessel-Gauss beams, the beam parameter is very small but cannot be zero. Similarly, for NDSTPs, the beam parameter is rapidly decreasing with decreasing q_3 (see Supplementary Fig. 2) with pulses becoming nondiffracting at q_3=1.

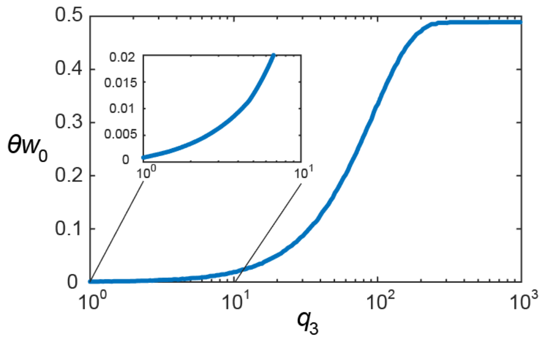

Supplementary Figure 2 Beam parameter of NDSTPs as a function of q_3, where θ is the divergence angle and w_0 is the waist defined as the FWHM of the pulse at focus ($z=0, t=0$).

Note 3: Topological numbers of skyrmions.

Topological properties of a skyrmionic configuration can be characterized by the skyrmion number [4]:

$$\begin{aligned} s &= \frac{1}{4\pi}\iint_{\sigma} \mathbf{n}\cdot\left(\frac{\partial \mathbf{n}}{\partial x}\times\frac{\partial \mathbf{n}}{\partial y}\right)\mathrm{d}x\mathrm{d}y s = \frac{1}{4\pi}\int_0^{r_\sigma}\mathrm{d}r\int_0^{2\pi}\mathrm{d}\theta\frac{\mathrm{d}\beta(r)}{\mathrm{d}r}\frac{\mathrm{d}\alpha(\theta)}{\mathrm{d}\theta}\sin\beta(r) \\ &= \frac{1}{4\pi}[\cos\beta(r)]_{r=0}^{r=r_\sigma}[\alpha(\theta)]_{\theta=0}^{\theta=2\pi} = p\cdot m \end{aligned} \tag{S26}$$

where $\mathbf{n}(x,y)$ represents the vector field to construct a skyrmion and σ the region to confine the skyrmion, which can be infinity (for an isolated skyrmion) also can be a cell of a periodic distribution (for skyrmion lattices). The skyrmion number is an integer counting how many times the vector $\mathbf{n}(x,y)=\mathbf{n}(r\cos\theta, r\sin\theta)$ wraps around the unit sphere, as the mapping shown in Supplementary Fig. 3(a). For mapping to the unit sphere, the vector can be given by $\mathbf{n}=(\cos\alpha(\theta)\sin\beta(r), \sin\alpha(\theta)\sin\beta(r), \cos\beta(r))$. The skyrmion number can be separated into two integers: the polarity, $p=\frac{1}{2}[\cos\beta(r)]_{r=0}^{r=r_\sigma}$, means that the vector direction is down (up) at center $r=0$ and up (down) at boundary $r\to r_\sigma$ for $p=1$ ($p=-1$), and the vorticity, $m=\frac{1}{2\pi}[\alpha(\theta)]_{\theta=0}^{\theta=2\pi}$, controls distribution of the transverse field components. In the case of a helical distribution, an initial phase γ should be added, $\alpha(\theta)=m\theta+\gamma$. Supplementary Fig. 3(b-g) show the theoretical results of selective topological structures of skyrmions with various values of polarity, vorticity, and helicity, (p, m, γ).

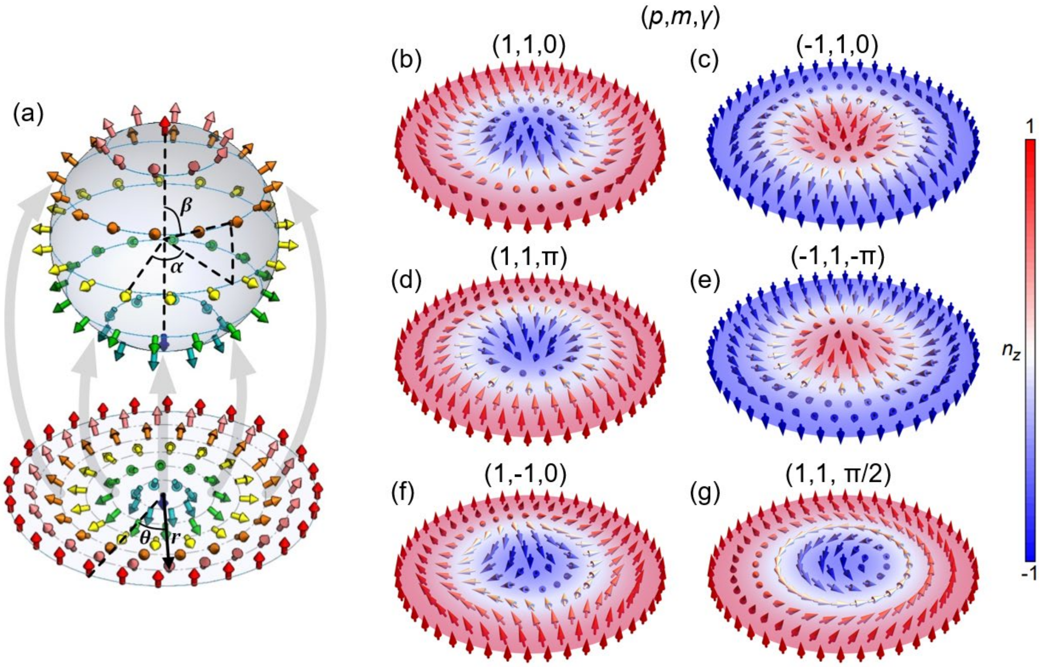

Supplementary Figure 3 (a) Representations of unit sphere wrapping for a skyrmion. (b-g) theoretical results of selective topological structures of skyrmions with various values of (p, m, γ) = (1,1,0), (-1,1,0), (1,1,π), (-1,1,-π), (1,-1,0), and (1,1, π/2), respectively.

Note 4: Plane wave expansion.

The spectrum of a supertoroidal light pulse, given by $E(r, z, t)$, can be directly obtained by Fourier transform from the space-time (r, z, t) to the wavevector-frequency domain (k_r, k_z, ω) [5]:

$$\tilde{E}(k_r, k_z, \omega) = \int_{-\infty}^{\infty}\int_{-\infty}^{\infty}\int_{-\infty}^{\infty} E(r, z, t) \exp[-i(k_r r + k_z z + \omega t)]\, drdzdt \tag{S27}$$

Limited by the solution of Maxwell's equations, the entire spectrum of a space-time light pulse must be distributed on the surface of light cone, i.e the conic surface with unit slope of its generatrix in the coordinate $(k_r, k_z, \omega/c)$. The numerically evaluated results for selected toroidal, supertoroidal, and nondiffracting supertoroidal pulses with $q_2=100q_1$ are shown in Supplementary Fig. 4. In all cases, we observe that the energy of the forward propagating components is over 99% of the total pulse energy. Especially, when the value of q_3 is decreased, the spectrum will approach a thin line along a conic section. If we further increase the parameter of q_2, the spectral line will become further thinner approaching the ideal infinitely thin line, corresponding to the case of exact nondiffraction.

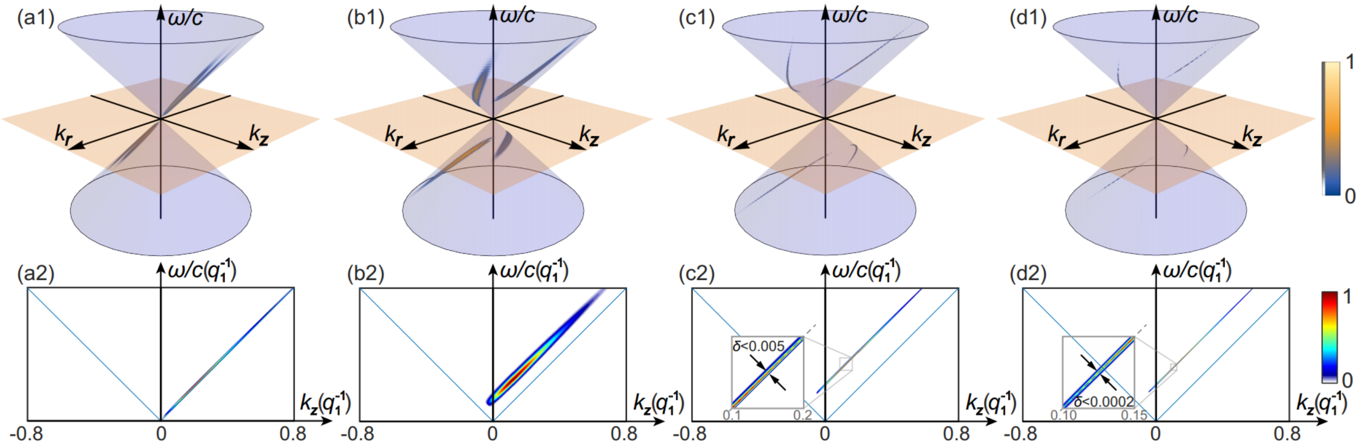

Supplementary Figure 4 The power spectrum distributions $\left|\tilde{E}(k_r, k_z, \omega)\right|^2$ on light cone via plane wave decomposition of toroidal (a1,a2), supertoroidal (b1,b2), and nondiffracting supertoroidal (c1,c2) pulses in the $(k_r, k_z, \omega/c)$ domain (a1-c1), and their projections onto the k_z–ω/c plane (a2-c2). The blue surface in the top row of graphs corresponds to the light cone. The following pulse parameters were used: (a1,a2) $q_2=100q_1$, $q_3=\infty$, $\alpha=1$; (b1,b2) $q_2=100q_1$, $q_3=\infty$, $\alpha=50$; (c1,c2) $q_2=100q_1$, $q_3=q_1$, $\alpha=1$; (d1,d2) $q_2=500q_1$, $q_3=q_1$, $\alpha=1$;

The plane wave expansion of NDSTPs allows us to study quantitatively the effects of finite apertures on the pulse propagation. We simulated the propagation of nondiffracting supertoroidal pulses (q_1=1μm, q_2=200μm, q_3=1μm, α=1) following truncation at focus by a finite circular aperture (see Supplementary Fig. 4) using plane wave expansion methods [6]. We considered apertures with diameter, d, between 0.5 mm and 0.05 mm and calculated the electric field intensity integrated over the duration of the pulse as a function of propagation distance (see Supplementary Fig. 5(a-e) for characteristic cases). We quantified the effect of the aperture by calculating the propagation distance, L, at which the pulse FWHM increases by 1%. The dependence of L on the aperture size, d, is presented in Supplementary Fig. 6. As the aperture size decreases from 0.5 mm to 0.05 mm, the propagation distance L decreases from ~148 mm to ~7 mm, respectively. The results presented here are comparable to those obtained for different types of nondiffracting beams in the optical [7,8] and microwave [9] parts of the spectrum and illustrate the practicality of the experimental generation of NDSTPs.

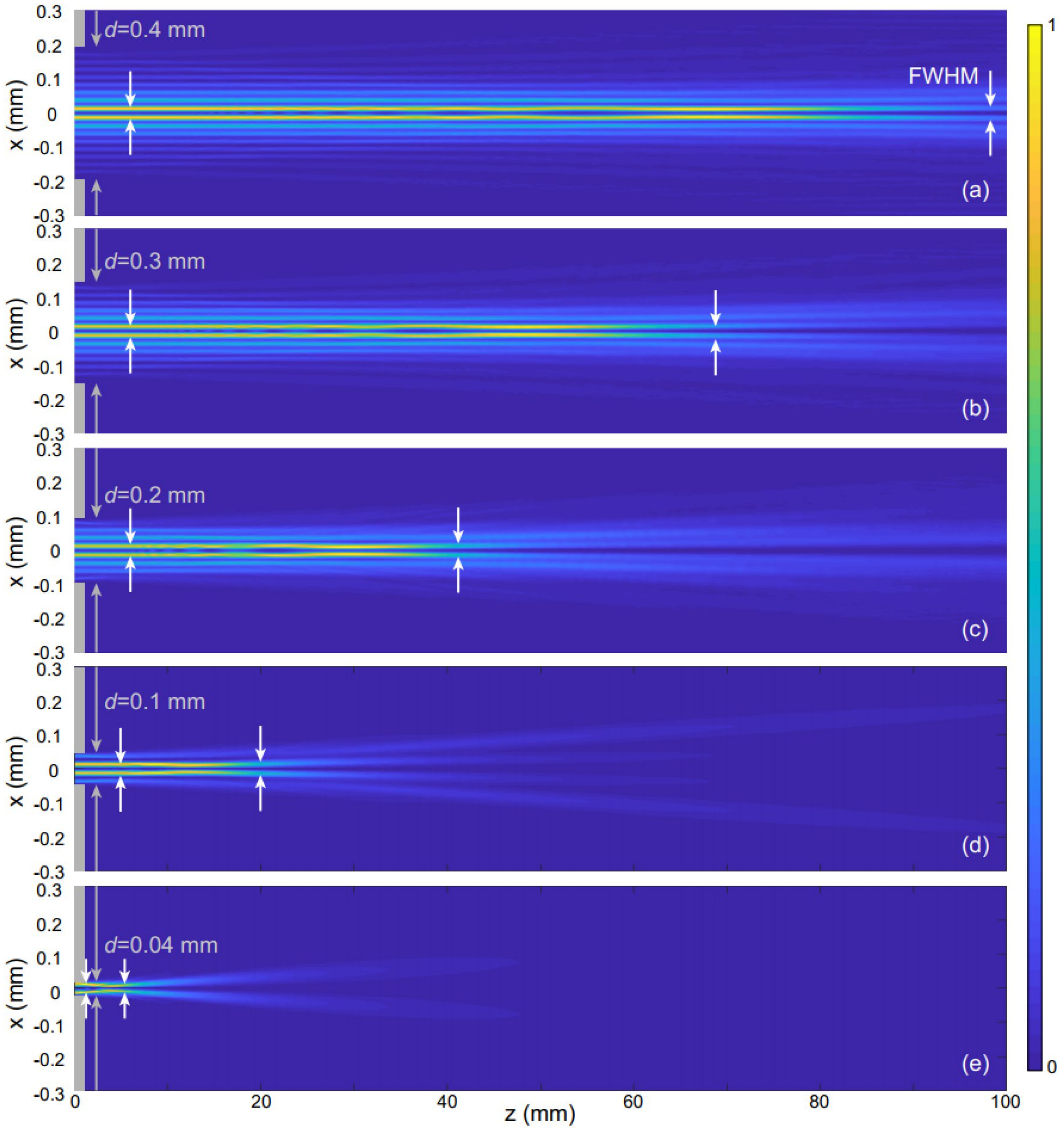

Supplementary Figure 5 The simulated results of the evolution of integrated intensity distribution upon propagation, $\int|E(r,z,t)|^2dt$, of the nondiffracting supertoroidal pulse (q_1=1μm, q_2=200μm, q_3=1μm, α=1) over a distance of 100 mm under the truncation of practical aperture with diameter, d, of (a) 0.4 mm, (b) 0.3 mm, (c) 0.2 mm, (d) 0.1 mm, and (e) 0.04 mm, respectively. The white arrows indicate the propagation distance at which the FWHM of the pulse increases by 1%.

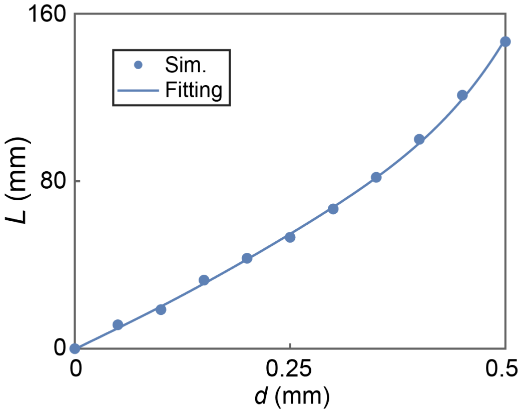

Supplementary Figure 6 Non-diffracting propagation distance, *L*, vs diameter of the aperture, *d*.

References

[1] R.W. Ziolkowski, *Phys. Rev. A* **39**, 2005 (1989).

[2] R. W. Hellwarth and P. Nouchi, *Phys. Rev. A* **54**, 889 (1996).

[3] Y. Shen, Y. Hou, N. Papasimakis and N. I. Zheludev, *Nat. Commun.* **12**, 5891 (2021); Supplementary Note 1.

[4] Y. Shen, *Opt. Lett.* **46**, 3737–3740 (2021).

[5] A. Zdagkas, et al., *Phys. Rev. A* **102**, 063512 (2020).

[6] M. Born and E. Wolf. Principles of optics: electromagnetic theory of propagation, interference and diffraction of light. Elsevier, 2013.

[7] H. E. Kondakci and A. F. Abouraddy, *Nat. Photonics* **11**, 733–740 (2017).

[8] L. Stoyanov, M. Zhekova, A. Stefanov, I. Stefanov, G. G. Paulus and A. Dreischuh, *Sci. Rep.* **10**, 21981 (2020).

[9] D. Comite, W. Fuscaldo, S. C. Pavone, et. al., *Appl. Phys. Lett.* 110, 114102 (2017).
