# Supplementary material for: Nondiffracting supertoroidal pulses and optical “Kármán vortex streets”
Source: Nat Commun. 2024 Jun 7;15:4863. doi: 10.1038/s41467-024-48927-5 (PMC11161654; doi:10.1038/s41467-024-48927-5)
Supplement: Supplementary file 3 — Description of Additional Supplementary Files [file 41467_2024_48927_MOESM3_ESM.pdf]

Description of Additional Supplementary Files

File Name: Supplementary Video 1
Description: Dynamic spatiotemporal evolutions of toroidal pulse, weakly-diffracting and nondiffracting supertoroidal pulses

File Name: Supplementary Video 2
Description: Singular electric field structure evolutions of toroidal pulse, weakly-diffracting and nondiffracting supertoroidal pulses

File Name: Supplementary Video 3
Description: Electromagnetic skyrmions and their evolutions in nondiffracting supertoroidal pulses
